# Chiral Metasurface for Near-Field Imaging and Far-Field Holography Based on Deep Learning

**DOI:** 10.3390/mi14040789

**Published:** 2023-03-31

**Authors:** Yihang Qiu, Sixue Chen, Zheyu Hou, Jingjing Wang, Jian Shen, Chaoyang Li

**Affiliations:** 1 School of Information and Communication Engineering, Hainan University, Haikou 570228, China; 2 State Key Laboratory of Marine Resource Utilization in South China Sea, Hainan University, Haikou 570228, China

**Keywords:** chiral metasurface, circular dichroism, deep learning, inverse design

## Abstract

Chiral metasurfaces have great influence on the development of holography. Nonetheless, it is still challenging to design chiral metasurface structures on demand. As a machine learning method, deep learning has been applied to design metasurface in recent years. This work uses a deep neural network with a mean absolute error (MAE) of 0.03 to inverse design chiral metasurface. With the help of this approach, a chiral metasurface with circular dichroism (CD) values higher than 0.4 is designed. The static chirality of the metasurface and the hologram with an image distance of 3000 μm are characterized. The imaging results are clearly visible and demonstrate the feasibility of our inverse design approach.

## 1. Introduction

As a particular geometric property, chirality is often used to characterize a class of material structures that cannot completely overlap with their mirror image, whether by translation or rotation. Substances with chiral characteristics, such as sugar molecules, DNA, amino acids, proteins, etc., are widely found in the organic world [1,2,3,4]. Left-handed circularly polarized light (LCP) and right-handed circularly polarized light (RCP) are two spins of the angular momentum of circularly polarized light. They have different responses when interacting with chiral molecules. This optical phenomenon is usually defined as circular dichroism (CD) or optical activity (OA). Circular dichroism refers to the dichroism of circularly polarized light and the differential absorption of LCP and RCP by substances. The property of polarized light that changes polarization direction when it passes through a substance is called optical activity. Although materials with chiral characteristics exist in nature, the chiral response of materials in nature is weak. As artificially designed structures, chiral metamaterials have stronger chirality than natural materials [5,6]. However, the complex process of three-dimensional (3D) structures limits the development of metamaterials [7,8]. The planar structural properties of the chiral metasurface overcome the above challenges [9]. Chiral metasurfaces have great potential to be used in practical applications, such as holographic imaging [10,11], biosensing [12,13], and vortex focusing [14].

Holography, as a 3D imaging technique, was first proposed by Gabor [15]. The hologram is generated by the interference between the reference beam and the target beam. Holography is capable of storing the amplitude and phase of the light field, as well as of reproducing 3D scenes, and reconstructing the image at the imaging location when a reference beam is shone on the holographic plate. However, conventional spatial light modulator (SLM) devices with large sizes, low resolutions, and minor fields of view (FOV) limit the development of holography. Due to the sub-wavelength unit of the metasurface, the resolution and quality of the image can be improved by reconstructing the image through the metasurface [11].

Although the study of the metasurface is promising, the on-demand design of the metasurface remains a challenge. Researchers must repeat many parametric scans and numerical calculations to find the metasurface that achieves the target effect. This process consumes a lot of time and computational resources. Therefore, there is an urgent need to find a way to design metasurfaces on demand. The inverse design process is usually guided by optimization algorithms: topology optimization, level set methods, genetic algorithms, etc. [16,17,18,19]. Their stochastic search nature severely limits the capabilities of the above inverse design algorithms. Therefore, as the size and complexity of the problem grow, these algorithms are inadequate for complex designs with multiple constraints.

As an interdisciplinary discipline in computing, deep learning spans many domains [20]. It can simulate various human learning processes to acquire new skills and knowledge. Deep learning can be trained using a small amount of data to find correlations between the data and, ultimately, to classify the data. There have been many studies on combining deep learning and metasurfaces that have significantly reduced the design time and improved the design efficiency of the metasurface [21,22,23,24,25].

We propose an efficient deep learning-assisted method for designing metasurfaces. According to this approach, only 750 sets of data are used for neural network model training, and the trained network model can obtain the target parameters on demand. The results show that the method enables the network to achieve an MAE of 0.03, which is 10% lower than that of CGAN [26], and shows good generalization and robustness. By training the network model to guide the design of the metasurface structure, the design cycle can be significantly shortened while the design efficiency is improved dramatically. An H-structured metasurface with a chiral optical response that is capable of reaching a 0.401 circular dichroism is proposed. The static chiral properties are shown based on the above metasurface mirror structure. This paper proposes phase-only modulated metasurface holograms with “HN” characters and shows the effect of the image at different imaging distances. The imaging results are within our expectations and demonstrate the feasibility of our proposed inverse design approach.

## 2. Materials and Methods

### 2.1. Metasurface Structure

**Figure 1:**
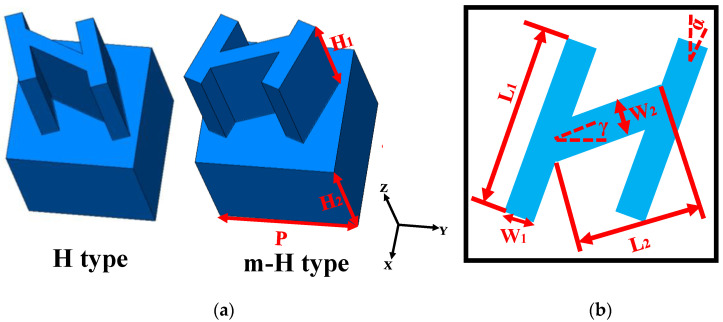
(**a**) The 3D structure of our proposed chiral metasurface; (**b**) the structural parameters.

Highly resistive silicon (Si) with a dielectric constant of e = 11.9 was used as the material for designing a metasurface model with shape H and mirror H in Figure 1a. The substrate height of this model was H_1_ = 200 μm. The width of the substrate was *p* = 180 μm, and the height of the H-shaped silicon column was H_2_ = 200 μm. As shown in Figure 1b, the deflection angle of the rectangular pillars on both sides of the H-shaped structure was α, the length is L_1_, and the width was W_1_ = 25 μm. The deflection angle of the pillar in the center concerning the horizontal line was γ, the length was L_2_, and the width was W_2_ = 20 μm. Periodic boundary conditions are set in the x and y directions, and in the z direction, there are open boundary conditions. Four parameters were scanned, L_1_, L_2_, α, γ. The range of L_1_ = (60, 100) μm, that of L_2_ = (50, 100) μm, that of α = (−20, 20) degree, and that of γ = (−20, 20) degree; and finally, a total of 750 sets of data were extracted.

### 2.2. Principles and Formula

With line polarization (LP) for incidence, the transmission coefficients of linearly and circularly polarized waves in the terahertz band are related as follows:

(1) $$\left(\begin{array}{cc} T_{RR} & T_{RL}\\ T_{LR} & T_{LL} \end{array}\right)=\frac{1}{2}\left(\begin{array}{cc} T_{\mathrm{xx}}+T_{yy}+i(T_{xy}-T_{yx}) & T_{\mathrm{xx}}-T_{yy}-i(T_{xy}+T_{yx})\\ T_{\mathrm{xx}}-T_{yy}+i(T_{xy}+T_{yx}) & T_{\mathrm{xx}}+T_{yy}-i(T_{xy}-T_{yx}) \end{array}\right)$$

*T_ij_* denotes the i-polarized transmitted electric field component in response to a j-polarized incident electric field of amplitude 1. R and L correspond to right circularly polarized (RCP) and left circularly polarized (LCP) waves, respectively. The CD is defined as follows:

(2) $$T_{CD}={\left|T_{LL}\right|}^{2}+{\left|T_{RL}\right|}^{2}-{\left|T_{RR}\right|}^{2}-{\left|T_{LR}\right|}^{2}$$

In addition, the strength of circular dichroism can be expressed in terms of the magnitude of ellipticity. The ellipticity can be obtained from the following equation:

(3) $$\eta =\frac{1}{2}\arctan(\frac{{\left|T_{LL}\right|}^{2}-{\left|T_{RR}\right|}^{2}}{{\left|T_{LL}\right|}^{2}+{\left|T_{RR}\right|}^{2}})$$

To design the target hologram, the Rayleigh–Sommerfeld (RS) diffraction formulation was used to obtain the holographic plate’s phase distribution and the imaging plane’s electric field distribution.

(4) $$U(x,y)=\frac{1}{i\lambda }\iint U_{0}(x_{0},y_{0})\frac{\exp(-ikr)}{r}\cos\theta dx_{0}dy_{0}$$

where (x,y) is any point on the image plane; (*x*_0_*,y*_0_) is any point on the metasurface; U (x,y) and U_0_(x,y) are the electric fields on the image plane and the metasurface; the wavelength in vacuum is *λ*; *z* is the imaging distance; $r=\sqrt{{\left(x_{0}-x\right)}^{2}+{\left(y_{0}-y\right)}^{2}}$; and the inclination factor is cosθ=z/r. For the inverse process, the electric field of the hologram is related to the electric field on the holographic plate as follows:

(5) $$U_{0}(x_{0},y_{0})=\frac{1}{i\lambda }\iint U(x,y)\frac{\exp(ikr)}{r}\cos\theta dx_{0}dy_{0}$$

In finding the optimal solution, to keep the generator and discriminator well synchronized, the output results were fed into the simulation software when using CGAN for inverse design. The formula for CGAN is shown below:

(6) $$\underset{G}{\min}\underset{D}{\max}V(D,G)=E_{x\sim p_{data}(x)}\left[\log(D(x|y))\right]+E_{z\sim p_{z}(z)}\left[\log(1-D(G(z|y)|y))\right]$$

**Figure 2:**
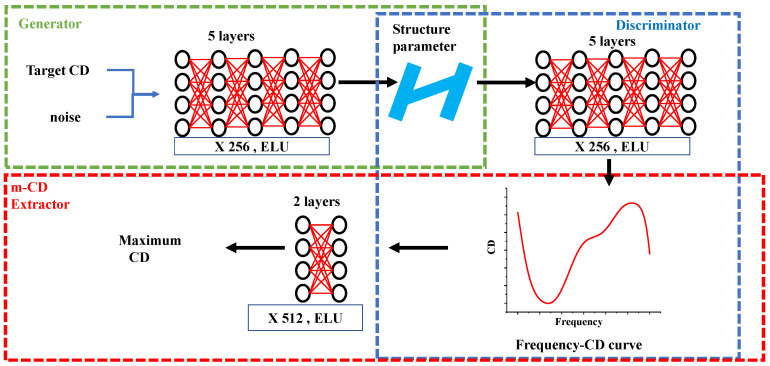
The hidden layers of the generator and discriminator are five layers with 256 nodes in each layer. The hidden layer of the max CD extractor is two layers with 512 nodes in each layer, and they are both composed in a fully connected manner.

This increases the difficulty of training, so it is not easy to visually verify the effect of the network training results when directly using the network of traditional CGAN for model training. A separate Conditional Generative Adversarial Network (sCGAN) training method is proposed, as shown in Figure 2.

The network consists of a generator, a discriminator, and a maximum CD extractor. Based on the idea of GAN, the discriminator’s input and output are changed. The method of directly inputting the metasurface structure parameters into the discriminator and obtaining the maximum CD has errors. This part of the network is divided into the discriminator and the maximum CD extractor to reduce the error. First, the CDs of all frequencies on the structure are obtained by the discriminator, and then the maximum value of the CD is received by the maximum CD extractor. This method changes the weights in the network to reduce the error. We train the discriminator and the maximum CD extractor by supervised learning. Until two sub-networks are trained as expected, the weights of the discriminator and the maximum CD extractor are kept constant. Then, combining the two networks, the generator is guided. Once the generator is trained by the discriminator and the maximum CD extractor as expected, we can use the generator alone to obtain the structural parameters with the target CD. A maximum CD and a set of random noises are used as input to the generator. The addition of random noise can make the output results have multiple sets of different parameters that satisfy the design requirements. This method can replace the time-consuming process of the simulation software. The calculation of circular dichroism by the simulation software takes about 1 h, while prediction by this network model takes less than 1 s.

Since the generator input is CD with a value range from 0 to 1, the output is a structural parameter. If the dimensions of the two are different, this can cause the error. In addition, too large or too small input values can also lead to errors. The input data need to be pre-processed to avoid errors due to the input of too large or too small values into the network. We use the normalization method to scale the original input data to keep the input and output dimensions consistent. The input data are scaled in the range (0, 1), which can be expressed as follows:

(7) $$x=\frac{x_{0}-\min x_{0}}{\max x_{0}-\min x_{0}}$$

*x*_0_ is the original data in the sample, and *x* is the normalized value. After the normalization operation, we can reduce the error due to the difference in the data input dimensions. When using the network for backward design, we can recover the output values using the following:

(8) $$x_{0}=x(\max x_{0}-\min x_{0})+\min x_{0}$$

In order to address the highly nonlinear problems and gradient vanishing, the ELU function is used as an activation function. Its corresponding equation is shown below:

(9) $$f(x_{0})=\{\begin{array}{c} x,x\geq 0\\ \alpha (e^{x}-1),x<0 \end{array}$$

The original input of the function is *x*, and the range of α is (0,1). The ELU function incorporates the sigmoid and ReLU functions, changing the nonlinear representation of each network level. Making the average value of activation close to 0 can make learning faster. Its gradient is closer to the natural gradient and converges faster and more accurately. The BN layer is used to optimize the weights of each network layer to optimize the output. The following equation can express the output of the kth layer:

(10) $$y_{k}=\gamma \frac{x_{k}-\mu }{\sigma }+\beta$$

β and γ are the learnable hyperparameters of the BN layer, which can reconstruct the feature distribution to be learned by the original network. Here, μ is the mean of the kth layer sample and σ is the sample variance.

## 3. Results

### 3.1. Deep Learning Inverse Design of Metasurface Structure

**Figure 3:**
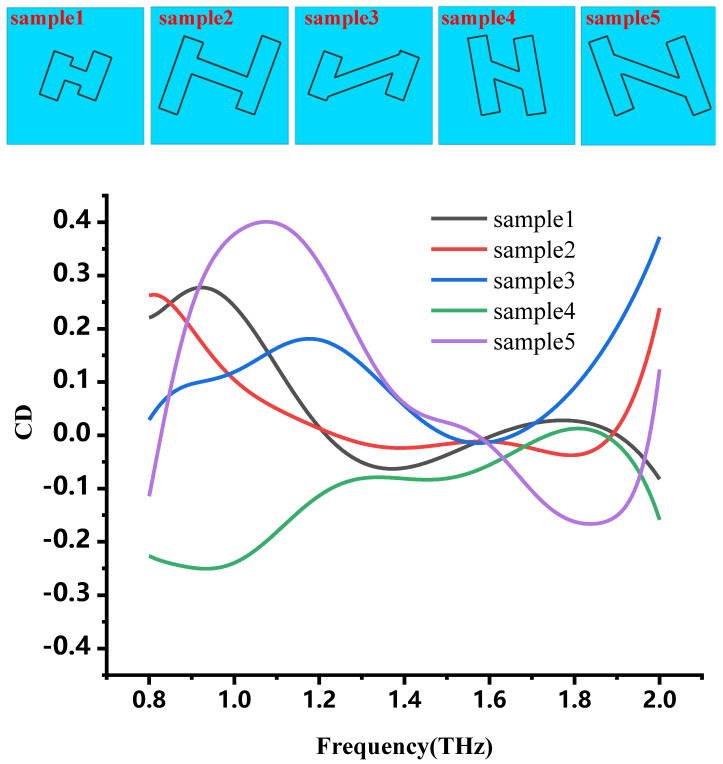
Plots of some samples and their circular dichroism curves for the data set of group 750.

We obtained a data set of 750 simulations. The circular dichroism curves and structures of some samples are shown in Figure 3 and Table 1. We divided the dataset into a training set and a test set with a ratio of 24:1.

**Figure 4:**
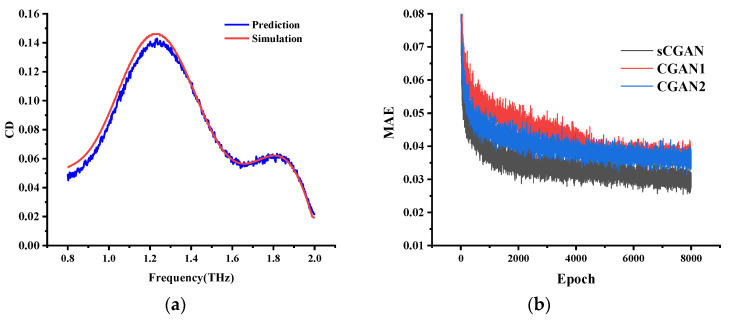
(**a**) Discriminator training results; (**b**) generator MAE for CGAN and sCGAN.

The training data set was fed into the discriminator and max CD extractor. When the discriminator training was completed, we randomly used a test set of data to validate the discriminator, and the results are shown in Figure 4a. The curves of the original data and the predicted curves are similar. There is some mismatch between the predicted and simulated CD in some of the frequency bands. The error is within an acceptable range, showing that the discriminator network was able to simulate the correspondence between the structural parameters of the metasurface and their corresponding CD curves. We fed the data set into the max CD extractor similarly, and after 10,000 iterations, the MAE reaches 0.0017. Then, the discriminator was connected with the max CD extractor and the generator, and the weights of the first two networks were set to be unchangeable in order to guide the training of the generator. Finally, we fed random noise and structural parameters together into the generator for 8000 iterations to make the MAE of the generator 0.03. Meanwhile, we trained the conventional CGAN model in the same way. sCGAN’s MAE was compared with conventional CGAN (without dividing the latter part of the network into a discriminator and a maximum CD extractor). The results are shown in Figure 4b.When the generator training was completed, we used the generator alone to perform the inverse design. We input a target CD of 0.41 and obtained multiple sets of structural parameters that satisfied the target and achieved our expected results.

**Figure 5:**
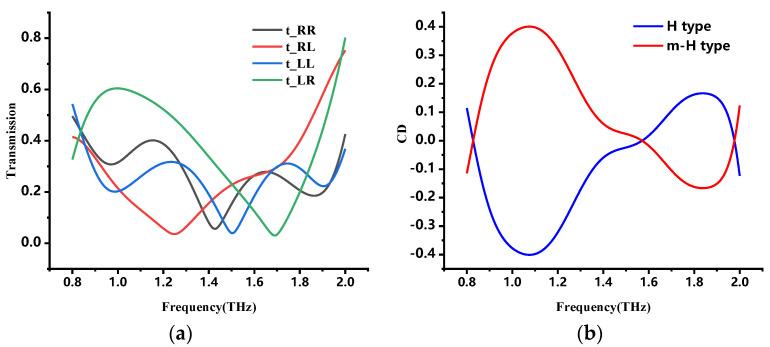
(**a**) Circular polarization transmission coefficient of H-type structure. (**b**) CD spectra of H type and m-H type.

The characteristic transmission curves of the inverse-designed structure are shown in Figure 5a. T_LR_ is higher than the other three sets of curves at around 1.07 THz. As shown in Figure 5b, the CD at 1.07 THz has a maximum value of 0.401. Meanwhile, due to the chirality of the metasurface unit, the two structures have opposite spectra. In addition, the CD of the m-H type structure with mirror symmetry in the *xoz* axis reaches 0.401 at 1.07 THz.

**Figure 6:**
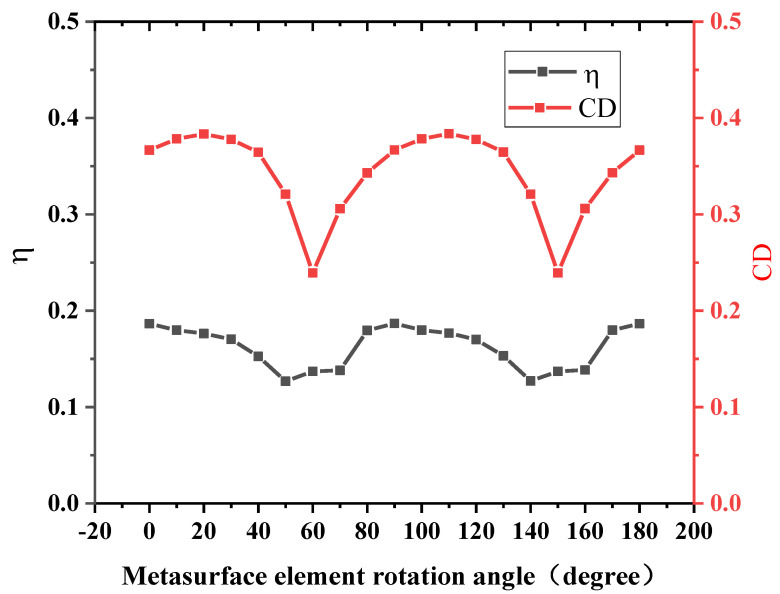
The trend of ellipticity and circular dichroism for large-angle rotations of the metasurface.

The trends of ellipticity and circular dichroism in the case of metasurface cell rotation are shown in Figure 6. It can be seen that the circular dichroism and ellipticity change as the rotation angle of the metasurface unit changes. Moreover, the trends of circular dichroism and ellipticity are positively correlated.

### 3.2. Near-Field and Holographic Imaging

**Figure 7:**
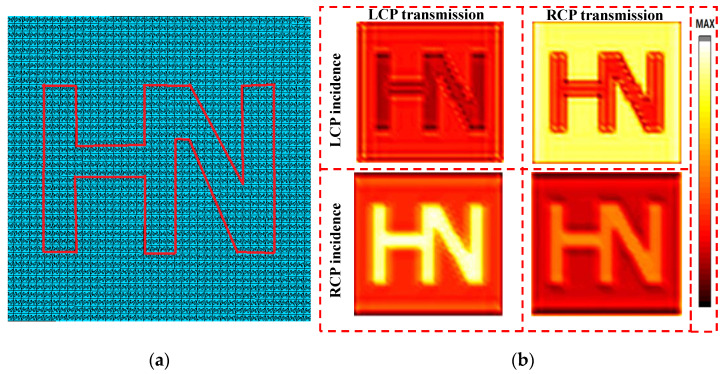
(**a**) The configuration of HN letters. (**b**) Cross-polarization and co-polarization images under LCP and RCP incidence. The detection probe is located at 200 μm from the array surface.

To detect the circular dichroism of the inverse-designed chiral metasurface, a set of inverse-designed structures and their mirror structures was chosen for the near-field imaging design of the metasurface. Both structures were used to construct the pattern of the letter HN and to detect the transmission of cross-polarization and co-polarization through the case of LCP and RCP incidence. Figure 7a shows the structure of the array, which consists of 40×40 metasurface cells. Near-field images of co-polarization and cross-polarization for LCP and RCP incidence are shown in Figure 7b. The cross-polarization image of the LCP and RCP incident is complementary, which verifies the metasurface structure’s static chiral properties.

**Figure 8:**
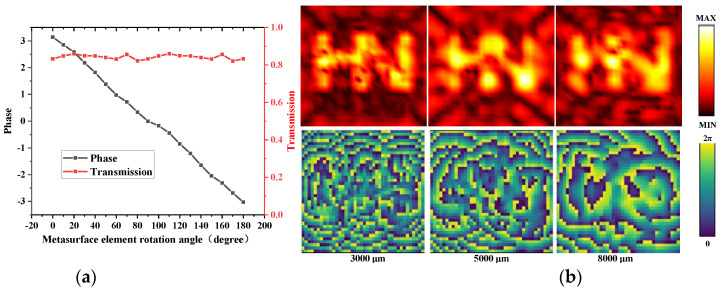
(**a**) The relationship between the rotation angle of the metasurface and phase. (**b**) The phase distribution on the metasurface hologram and holographic plate at 3000 μm, 5000 μm and 8000 μm imaging distances.

The relationship between the rotation angle of the metasurface unit and the phase is calculated, as shown in Figure 8a. Based on the RS diffraction formula, the holographic plate is designed for the desired image. Hologram and holographic plate phase distributions at different imaging distances are shown in Figure 8b.

The hologram is clearly at the image distance of 3000 μm, and the image quality decreases gradually with the increase in the imaging distance. The theoretical calculation assumes that the electric field intensity on the metasurface is a constant value. Still, in practice, the electric field intensity on the metasurface changes as the metasurface cell rotates in Figure 8a. Therefore, there is an error in the electric field on the imaging surface. In addition, there is some noise in the image. These noises appear due to the incident of circularly polarized light. When detecting the co-polarization transmission, the incident wave affects the detection results in the direction of co-polarization. Despite the effect of noise, the quality of the holograms follows our expectations. Meanwhile, near-field imaging and far-field holography results show that the method of inverse engineering the metasurface by deep learning is somewhat feasible.

## 4. Discussion

In this paper, we propose an efficient method for inverse designing a metasurface. Based on the CGAN model, a new max CD extractor is introduced to optimize the data processing, and the supervised learning CGAN is transformed into unsupervised learning sCGAN. An H-type chiral metasurface structure possessing 0.401 circular dichroism at 1.07 THz is obtained by predicting the target CD. In addition, our imaging results reveal the feasibility of this inverse design method. We verify the static chiral properties of the metasurface through the near-field image. We modulated the electric field intensity distribution on the imaging surface by changing the phase on the holographic plate according to the RS formula. We achieved a clear hologram at an imaging distance of 3000 μm. In addition to near-field and holographic imaging, our on-demand design method has many applications, such as in vortex metasurfaces, focusing metasurfaces, metalens, etc. This design method will substantially reduce the consumption of human and computational resources in designing the metasurface. 

## Figures and Tables

**Table 1:** Sample structure parameter.

	α (Degree)	γ (Degree)	L_1_ (μm)	L_2_ (μm)
sample 1	−20	−20	60	50
sample 2	−20	−20	100	100
sample 3	−20	20	60	100
sample 4	10	−20	100	60
sample 5	20	−20	100	100

## Data Availability

Research data presented in this study are available on request from the corresponding author.
